# Active Targeted Nanoemulsions for Repurposing of Tegaserod in Alzheimer’s Disease Treatment

**DOI:** 10.3390/pharmaceutics13101626

**Published:** 2021-10-06

**Authors:** Line Séguy, Léna Guyon, Manon Maurel, Pascal Verdié, Audrey Davis, Sophie Corvaisier, Vincent Lisowski, Patrick Dallemagne, Anne-Claire Groo, Aurélie Malzert-Fréon

**Affiliations:** 1 Centre d’Etudes et de Recherche sur le Médicament de Normandie, Normandie University, Unicaen, CERMN, 14000 Caen, France; line.seguy@unicaen.fr (L.S.); lena.guyon@outlook.fr (L.G.); audrey.davis@unicaen.fr (A.D.); sophie.corvaisier@unicaen.fr (S.C.); patrick.dallemagne@unicaen.fr (P.D.); 2 Institut des Biomolécules Max Mousseron UMR 5247, Université de Montpellier, ENSCM, 34093 Montpellier, France; manon.maurel@umontpellier.fr (M.M.); pascal.verdie@umontpellier.fr (P.V.); vincent.lisowski@umontpellier.fr (V.L.)

**Keywords:** repurposing, Alzheimer’s disease, brain-targeting, nanoemulsions, peptide-22

## Abstract

Background and Purpose: The activation of 5-HT_4_ receptors with agonists has emerged as a valuable therapeutic strategy to treat Alzheimer’s disease (AD) by enhancing the nonamyloidogenic pathway. Here, the potential therapeutic effects of tegaserod, an effective agent for irritable bowel syndrome, were assessed for AD treatment. To envisage its efficient repurposing, tegaserod-loaded nanoemulsions were developed and functionalized by a blood–brain barrier shuttle peptide. Results: The butyrylcholinesterase inhibitory activity of tegaserod and its neuroprotective cellular effects were highlighted, confirming the interest of this pleiotropic drug for AD treatment. In regard to its drugability profile, and in order to limit its peripheral distribution after IV administration, its encapsulation into monodisperse lipid nanoemulsions (Tg-NEs) of about 50 nm, and with neutral zeta potential characteristics, was performed. The stability of the formulation in stock conditions at 4 °C and in blood biomimetic medium was established. The adsorption on Tg-NEs of peptide-22 was realized. The functionalized NEs were characterized by chromatographic methods (SEC and C_18_/HPLC) and isothermal titration calorimetry, attesting the efficiency of the adsorption. From in vitro assays, these nanocarriers appeared suitable for enabling tegaserod controlled release without hemolytic properties. Conclusion: The developed peptide-22 functionalized Tg-NEs appear as a valuable tool to allow exploration of the repurposed tegaserod in AD treatment in further preclinical studies.

## 1. Introduction

Neurodegenerative diseases are on the rise worldwide. In 2016, it was estimated that 43.8 million people lived with dementia [1], and this number is expected to grow with people aging. Alzheimer’s disease (AD) is the most common form of senile dementia, characterized by memory loss and other cognitive impairment with aging. The neuropathological hallmarks of AD include neuronal loss, synaptic dysfunction, intraneuronal neurofibrillary tangles composed of hyperphosphorylated and misfolded tau protein, and extracellular senile plaques, due to the accumulation and the aggregation of β-amyloid peptides (Aβ) [2]. This peptide is a product of the proteolytic degradation of the amyloid precursor protein (APP) according to the amyloidogenic pathway, which involves β- and γ-secretases. An alternative nonamyloidogenic APP cleavage involves α-secretase and leads to the release of the soluble sAPPα fragment, which has neurotrophic and neuroprotective properties [3]. In the last decade, the activation of serotonin receptors was identified to stimulate this nonamyloidogenic pathway, and thus to preclude Aβ formation [4]. The administration of a 5-hydroxytryptamine type-4 receptor (5-HT_4_R) agonist in a 5XFAD mouse model was reported to have beneficial effects on learning and memory loss linked with a reduction of the number of observed amyloid plaques [5,6]. In this purpose, serotonin receptors agonists emerged as novel opportunities in AD treatment. Several new structural 5-HT_4_R ligands or repurposed 5-HT_4_R agonists have recently been the subject of clinical trials for treating cognitive impairment [7].

Among 5-HT_4_R agonists to consider, tegaserod was approved by the Food and Drug Administration (FDA) in 2002, under the trade name Zelnorm^®^. This drug was available for oral use, as 6 mg tablets taken twice daily, as the first available therapy to treat irritable bowel syndrome with constipation as a main symptom (IBS-C) in women. Indeed, in gastrointestinal tract, the activation of 5-HT_4_R increases propulsive motility and visceral antinociceptive activity [8]. IBS-C syndrome is the most common gastrointestinal disorder, and it affects around 11% of the population worldwide [9]. However, a retrospective analysis of pooled clinical trials data reported a statistically significant imbalance in the number of ischemic cardiovascular events in patients taking Zelnorm^®^ compared to placebo [10]. This study led to the tegaserod market withdrawal in 2007, which was only maintained by the FDA as investigational drug with restricted use [11]. During the following eleven years, tegaserod was still studied to elucidate the mechanism of related cardiac events, but this study remains unknown to date. While several studies failed to confirm the increased risk of cardiovascular outcomes in tegaserod users [12,13], the prior observation from 2007 cannot be ruled out. In 2019, after a complete safety review by the FDA, the reintroduction of Zelnorm^®^ was approved in 65-and-younger women patients with IBS-C and was contraindicated in patients with a history of myocardial infarction, stroke, transient ischemic attack or angina [14]. Furthermore, some recent studies showed that tegaserod has been repurposed as an antitumor drug to treat cancers. Its action mechanism would be independent of its original target 5-HT_4_R [15,16,17].

In 2009, Liu et al. reported the neurogenesis and neuroprotective properties of tegaserod after in vitro and in vivo experiments on the enteric nervous system [18]. In other studies, tegaserod appeared as a polysialic acid (PSA) mimetic compound with peripheral nerve regeneration activity [19,20].

However, so far, and to our knowledge, repurposing of tegaserod for AD treatment has never been reported. In consequence, from pharmacological assays, we propose to define the potential of tegaserod against AD. One main advantage of drug repurposing is that a lot of data have already been generated, especially in terms of safety, toxicity and pharmacokinetic profiles. However, because tegaserod may be considered as an “old drug” since first preclinical and clinical trials have been realized in the 1990s, we propose also to characterize with common current High-Throughput Screening (HTS) tools [21,22] the physicochemical properties of tegaserod that are determinants for its drugability profile (solubility, permeability, etc.).

Moreover, to succeed in drug repurposing, it is crucial to examine the best way to administer the drug in order to optimize the drug interaction with the new target. The formulation issue can also be advantageously integrated in the repurposing of the drug. If required, drug-delivery systems, such as nanocarriers, can be proposed to bypass drug low solubility, low permeability and/or low target specify issues (Seguy revue). Moreover, to ensure the best delivery to the new target, e.g., a limited peripheral distribution when a central drug accumulation is required in central nervous system (CNS)-pathologies treatment, functionalized drug-loaded nanocarriers can be developed. Indeed, thanks to their functionalization with targeting ligands, the nanocarriers become able to overcome some physiological barriers and to increase drug delivery into target tissues to treat without causing damage to healthy tissues [23].

To reach the brain in therapeutically relevant amounts, drugs or nanocarriers must cross a physiological barrier that hindered their delivery: the blood–brain barrier (BBB). Thanks to its composition, the BBB separates and protects the central nervous system from the bloodstream and maintains the brain homeostasis by controlling substances exchange between them.

Among the approaches studied to circumvent the BBB, those based on intracarotid injection of hyperosmotic compounds, such as mannitol [24,25,26,27,28,29], or on transient BBB opening by ultrasounds [30] are particularly invasive. An alternative to these strategies is cell-penetrating peptides (CPPs) or BBB shuttle peptides (BBBSP) to deliver drugs or nanovectors into the brain without effect on the BBB integrity. While CPPs suffer from lack of brain selectivity, BBBSP are ligands of receptors on the BBB, especially on the brain endothelium [31]. By targeting endogenous receptors, these peptides shuttle their cargo through the BBB by receptors-mediated transcytosis (RMT), using endogenous transport mechanisms for brain nutrients. RMT is one of the most commonly used and promising method to improve CNS drugs with modified nanocarriers. The receptors highly expressed at the BBB were widely characterized and studied as BBB transport receptors including transferrin (TfR), low-density lipoproteins (LDL-Rs), insulin and leptin receptors [32,33]. It is not surprising that LDL-Rs are the most exploited receptors to transport therapeutics across the BBB with peptides [31], as they have been shown to be a serious option in the field of RMT-based brain targeting to improve CNS drug delivery [34]. Various moieties or peptides derived from Apo-B and Apo-E have been proven their efficacy to improve the delivery of therapeutics to the CNS [35,36,37].

The purpose of this work is to carry tegaserod to the CNS after intravenous administration while avoiding any cardiac side events. In this study, we propose to develop tegaserod-loaded nanocarriers that could be functionalized by a BBB shuttle peptide.

**Figure 1:**
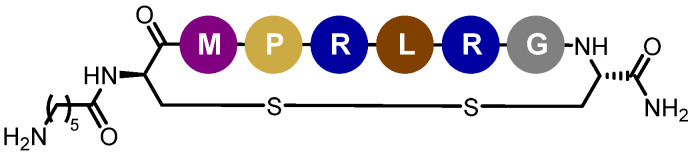
Structure of peptide-22.

On the basis of our previous formulation studies, the development and the pharmaceutical characterization of tegaserod-loaded lipid nanoemulsions will be realized [38]. Considering that the LDL-Rs is a relevant target to shuttle therapeutics into the CNS after receptor-mediated transcytosis, functionalization of the nanoemulsions will be attempted by a peptide ligand having an improved binding affinity for this receptor [39]. Peptide-22 is a cyclic peptide with reduced peptide bonds and one non-natural amino acid that exhibit a half-life of about 3 h and no competition with endogenous LDL [39] (Figure 1). From in vitro and in vivo studies, the interest in the surface modification of polymer nanoparticles or of liposomes by peptide-22 to facilitate BBB permeability and to improve therapeutic efficiency of carried anticancer drugs into the brain has already been demonstrated [40,41]. However, to date, no lipid nanoemulsions functionalization, nor application to AD therapeutic tools, have been reported. Thus, the final aim of the present work is to functionalize and characterize by the most potent available techniques the peptide-22-functionalized tegaserod-loaded nanoemulsions to allow their further valuable preclinical evaluation.

## 2. Materials and Methods

### 2.1. Materials

Kolliphor^®^ HS 15 (macrogol 15 hydroxystearate) was a gift from BASF (Ludwigshafen, ermany). Labrafac^®^ WL 1349 and Transcutol^®^ HP were kindly provided by Gattefossé S.A. (Saint-Priest, France). Tegaserod was purchased from Synthenova SAS (Hérouville-Saint-Clair, France). The peptide-22 was synthesized by the peptide team of the Institut des Biomolécules Max Mousseron (IBMM, Montpellier, France) according to Malcor et al. [39]. Acetylthiocholine iodide (ATC), butyrylthiocholine iodide and 5,5-dithiobis-(2-nitrobenzoic) acid (DTNB), human acetylcholinesterase (AChE), equine butyrylcholinesterase (BuChE), donepezil, Triton^®^ X-100 (octylphenol PEG-10 ether), KH_2_PO_4_, KCl and NaH_2_PO_4_·H_2_O were purchased from Sigma-Aldrich (Saint-Quentin Fallavier, France). Tacrine was provided from Tocris (Lille, France). Prisma Buffer HT pH 7,4, sink brain buffer, corticosterone and theophylline was obtained from pION (Billerica, Etats-Unis, MA, USA). Methanol, acetonitrile, acetone, water of HPLC grade were purchased from Carlo Erba Reagents (Val de Reuil, France). Formic acid, Na_2_HPO_4_·12 H_2_O, Tris hydrochloride, HEPES and DMSO were obtained from Fisher Scientific (Illkirch, France). NaCl was provided by Acros Organics (Geel, Belgium).

### 2.2. Methods

#### 2.2.1. Tegaserod Characterization

##### In Vitro Measurement of AChE and BuChE Activity

The inhibitory capacity of tegaserod on acetylcholinesterase (AChE) and butyrylcholinesterase (BuChE) biological activity was evaluated through the use of the spectrometric method of Ellman [42]. Lyophilized BuChE from equine serum (eqBuChE) was dissolved in 0.1 M phosphate buffer (pH 7.4) to have enzyme solutions stock with 2.5 units/mL enzyme activity. AChE from human erythrocytes (hAChE, buffered aqueous solution, ≥500 units/mg protein (BCA) was diluted in 20 mM HEPES buffer pH 8, 0.1% Triton X-100 to have enzyme solution with 0.25 unit/mL enzyme activity. Tegaserod was dissolved at 5 · 10^−2^ M in analytical grade DMSO. Donepezil or tacrine was used as reference standards.

In the procedure, 100 µL of 0.3 mM DTNB that was dissolved in a phosphate buffer with pH 7.4 was added into the 96-well plates, followed by 50 µL of enzyme (0.05 U final) and 50 µL of the test compound solution diluted in phosphate buffer, corresponding to the final concentration of interest (typically, 10 µM). After 5 min of preincubation at 25 °C, the reaction was then initiated by the injection of 50 µL of 1 mM acetyl- or butyrylthiocholine iodide solution. The hydrolysis of acetyl- or butyrylthiocholine was monitored by the formation of yellow 5-thio-2-nitrobenzoate anion as the result of the reaction of DTNB with thiocholine, released by the enzymatic hydrolysis of acetyl- or butyrylthiocholine, at a wavelength of 412 nm, using a 96-well microplate plate reader (Synergy 2, Biotek, Colmar, France). The rate of absorbance increase at 412 nm was determined 4 min after addition of the acetyl- or butyrylthiocholine iodine solution. Assays were performed with a blank containing all components except acetyl- or butyrylthiocholine, to account for non-enzymatic reactions. The percent inhibition due to the presence of the test compound was calculated by the following expression: ((v_0_ − v_i_)/v_0_) 100, where v_i_ is the rate calculated in the presence of inhibitor and v_0_ is the enzyme activity. IC_50_ values were determined graphically by plotting the % inhibition versus the logarithm of various inhibitor concentrations in the assay solution, using the GraphPad Prism software (version 6.01, GraphPad Software, La Jolla, CA, USA). Experiments were performed in *n* = 3.

##### Effects of Tegaserod with Soluble Aβ Peptides in a Primary Culture of Hippocampal Neurons: Survival and Neurite Network Evaluation

Rat hippocampal neurons were cultured as described by Callizot et al. [43]. The culture medium was a Neurobasal medium with a 2% solution of B27 supplement, 2 mM L-glutamine, 2% of penicillin (10,000 U/mL) and streptomycin (10 mg/mL) solution, and 10 ng/mL of brain-derived neurotrophic factor (BDNF). Cells were seeded at a density of 20,000 cells per well in 96-well plates pre-coated with poly-l-lysine and cultured at 37 °C, in an air (95%)–CO_2_ (5%) incubator. The medium was changed every 2 days.

The hippocampal neurons were exposed with Aβ solutions after 17 days of culture. The Aβ_1–42_ was prepared as described by Callizot et al. [43]. Briefly, Aβ_1–42_ peptide was dissolved in the defined culture medium mentioned above, at an initial concentration of 40 μM. This solution was gently agitated for 3 days, at 37 °C, in the dark, and immediately used after being properly diluted at a final concentration of 15 µM in culture medium (=1.5 μM of soluble Aβ peptides, evaluated by automatic WB) in presence of compounds. Donepezil (1 μM) or tegaserod (0.5 nM to 1 μM) were solved in culture medium and preincubated 1 h before Aβ application. The assays were carried out in a 96-well plate (6 wells per condition).

24 h after intoxication, the hippocampal neurons were fixed by a cold solution of ethanol (95%) and acetic acid (5%) for 5 min at −20 °C. After permeabilization with 0.1% of saponin, cells will be incubated for 2 h with a chicken polyclonal antibody anti microtubule-associated-protein 2 (MAP-2, RRID:AB_2138153), diluted at 1/1000 in phosphate-buffered saline (PBS) containing 1% fetal calf serum and 0.1% of saponin. This MAP-2 antibody allows the specific staining of neuronal cell bodies and neurites, and allows the study of neuronal cell death and neurite network. Antibodies were revealed with Alexa Fluor 488 goat anti mouse IgG (RRID:AB_2532075) and Alexa Fluor 568 goat anti-chicken IgG (SAB4600084, Sigma-Aldrich) at 1/400 dilution in PBS containing 1% FCS, 0.1% saponin, for 1 h, at room temperature. For each condition, 30 pictures (representative of the whole well area) per well were taken by using ImageXpress (Molecular Devices, RRID:SCR_016654) at 20× magnification. All images were taken with the same acquisition parameters. Analyses were performed automatically by using Custom Module Editor (Molecular Devices). The following endpoints were assessed: total number of neurons (neuron survival, number of MAP-2 positive neurons) and neurite network (in μm of MAP-2 positive neurons).

##### hERG Inhibition Assay

The hERG channel binding was determined with the Predictor^TM^ hERG Fluorescence Polarization Assay (PV5665 ThermoFischer, Waltham, MA, USA). Briefly, reagents were thawed and then mixed by pipetting 20× with Predictor^TM^ hERG membranes. The fluorescent tracer was diluted at 4 nM in the Predictor^TM^ hERG buffer. Tegaserod was dissolved in DMSO at 10 mM and was then diluted from 0.4 µM to 0.04 nM in the Predictor^TM^ hERG buffer. The assay was performed in a 96-well Greiner microplate (675093 Dutscher). Then, 25 µL of each concentration of tegaserod was dispensed into the plate. After that, 50 µL of the Predictor^TM^ hERG membranes and 25 µL of the fluorescent tracer 4 nM were added. The plate was incubated at room temperature for 4 h. The fluorescence polarization measurements were made by using a Synergy 2 microplate reader (Biotek Instrument, Colmar, France). Both parallel and perpendicular fluorescence were measured by using 530/25 nm excitation and 590/35 nm emission filters with a 570 nm dichroic mirror. The gain was fixed at 110. A known hERG channel blocker (E-4031), provided by the kit, was assayed as positive reference. Both E-4031 and M1 were tested at final concentrations, ranging from 0.01 nM to 100 µM. IC_50_ values were determined graphically by plotting the % inhibition versus the logarithm of 9 M1 concentrations in the assay solution, using the GraphPad Prism software (version 6.01, GraphPad Software, La Jolla, CA, USA).

##### Thermodynamic Solubility Determination at pH 7.4

Thermodynamic solubility at pH 7.4 of tegaserod was determined according to a miniaturized shake-flask method (Organisation for Economic Cooperation and Development guideline n°105) [44]. Phosphate Buffer solution (pH 7.4, 10 mM, ionic strength 150 mM) was prepared from Na_2_HPO_4_, KH_2_PO_4_ and KCl. A total of 990 µL of buffer was put into a 5 mL glass tube. Then 10 µL of 50 mM tegaserod solution in DMSO was added to the buffer (*n* = 3). Then, glass tubes were briefly sonicated and shacked by inversion at room temperature for 24 h. Tube contents were transferred in microtubes and centrifuged at 12,225× *g* for 10 min. Then 100 µL of supernatant was mixed with 100 µL acetonitrile in a Greiner UV microplate. The solubility determination was realized by spectrophotometry (Infinite M200, Tecan, Männedorf, Switzerland) at 312 nm from calibration curve obtained from six standard tegaserod solutions in a 50:50 (*v*/*v*) mixture of buffer with acetonitrile/DMSO (99:1; *v*/*v*). Calibration curves were linear within the concentration ranges studied (10–400 µM) with R² > 0.99.

##### Blood-Brain Barrier (PAMPA-BBB) and Gastrointestinal Tract Parallel Artificial Membrane Permeability (PAMPA-GIT) Assays

The PAMPA-BBB and the PAMPA-GIT experiments were conducted by using the PAMPA Explorer Kit (Pion Inc., Billerica, MA, USA), according to manufacturer’s protocol. Briefly, tegaserod was first dissolved in DMSO at 20 or 10 mM, for PAMPA-BBB experiment and PAMPA-GIT, respectively. For both PAMPA types, each stock solution was then 1/200 (*v*/*v*) diluted in Prisma HT buffer pH 7.4 (pION), and also in Prisma HT buffer pH 6.0, and pH 5.0 for PAMPA-GIT experiments. Then, 200 µL of these solutions was added to the donor plates (*n* = 6 and *n* = 4 for PAMPA-BBB and PAMPA-GIT, respectively). Each membrane filter of the acceptor plate (P/N 110243) was coated with either 5 µL of BBB-1 Lipid (P/N 110672) or GIT-0 Lipid (P/N 110669). Each well of the acceptor plates was filled with either 200 µL of Brain Sink Buffer (P/N 110674) or Acceptor Sink Buffer (P/N 110139). In both experiments, after incubation at room temperature for 4 h without stirring, the sandwich was separated, and the amounts of drug in both donor and acceptor compartments were measured through UV–visible spectra by the microplate reader (Infinite M200, Tecan, Männedorf, Switzerland). The permeability value (Pe) was calculated by the PAMPA Explorer software version 3.7 (pION). For the PAMPA-BBB experiments, corticosterone (Pe = 134.7 ± 7.4 nm/s) and theophylline (Pe = 4.2 ± 0.2 nm/s) were used as references for high and low permeability, respectively. Control standards for PAMPA-GIT experiment were ketoprofen (with Pe of 648.1 ± 114.0 nm/s at pH 5.0; 317.6 ± 19.8 nm/s at pH 6.0; 43.5 ± 2.8 nm/s at pH 7.4) for pH dependent permeability and antipyrine (with Pe of 22.6 ± 0.5 nm/s at pH 5.0; 20.5 ± 5.1 nm/s at pH 6.0; 20.4 ± 0.1 nm/s at pH 7.4) for low permeability.

##### Calculated Physicochemical Properties of Tegaserod

Values of molecular weight, p*K*a, log*P* were calculated by using standard tools of the ChemAxon Package (Marvin 19.1.0, 2019, ChemAxon (http://www.chemaxon.com/, access date: 21 July 2021).

#### 2.2.2. Encapsulation of Tegaserod into Nanoemulsions

##### Nanoemulsions Formulation Process

Nanoemulsions were obtained according to the formulation process recently developed in our laboratory [38], based on spontaneous nanoemulsification. Briefly, Labrafac^®^ WL 1349, Kolliphor^®^ HS 15 and Transcutol^®^ HP were mixed under gentle magnetic stirring (250 rpm), heated to 70 °C and then cooled down to 25 °C. At this temperature, the magnetic stirring was increased from 250 to 750 rpm, and the aqueous phase (60 mM phosphate buffer, pH 7.2, 25 °C) was suddenly added to the anhydrous phase. Magnetic stirring was maintained for 15 min, at room temperature. The obtained formulation was filtered by using a 0.2 µm regenerated cellulose syringe filter (Minisart^®^ Syringe Filter, Sartorius, Goettingen, Germany). In the case of tegaserod-loaded nanoemulsions, tegaserod was weighed with anhydrous excipients and heated at 70 °C, under magnetic stirring (250 rpm). The anhydrous phase was ultrasonicated for 5 min, at room temperature. Then, nanoemulsions were prepared as previously described, with a phosphate buffer of 60 mM at pH 7.2.

##### Physicochemical Characterization of the Nanoemulsions

The average hydrodynamic diameter and the polydispersity index (PDI) were determined by dynamic light scattering (DLS), using a Zetasizer Ultra^®^ apparatus (Malvern Instruments, Worcestershire, UK) equipped with a 633 nm laser at a fixed scattering angle of 173°. Each analysis was carried out at 25 °C, and samples were diluted 1/100 (*v*/*v*) in 1 mM NaCl solution. Three consecutive measurements were performed for each characterization.

Zeta potential analyses were realized after dilution (1/100) in 1mM NaCl solution, using a NanoZS^®^ apparatus equipped with DTS 1070 cell. All measurements were performed in triplicate at 25 °C, with a dielectric constant of 78.5, a refractive index of 1.33, a viscosity of 0.8872 cP and a cell voltage of 150 V. The zeta potential was calculated from the mean electrophoretic mobility values, using the Smoluchowski equation.

The pH of nanoemulsions was measured by using a pH-meter (Eutech instrument, Landsmeer, Netherlands) equipped with a microprobe (Fisherbrand, Fisher Scientific, Illkirch, France). Osmolarity measurements were performed on a micro-osmometer autocal type 15/15M (Löser Messtechnik, Berlin, Germany) via the freezing-point method.

##### Stability Studies

The stability of tegaserod-loaded nanoemulsions was investigated over a storage period of 4 months at 4 °C. The formulations were stocked undiluted. The size distribution, zeta potential and drug payload were evaluated at regular intervals (1, 7, 14, 21, 28, 60 and 120 days).

The stability of tegaserod-loaded nanoemulsions was determined in PBS, pH 7.4 (European Pharmacopeia, 10th ed.), by incubating diluted nanoemulsions (1/100 and 1/500) at 37 °C during 24 h, under gentle horizontal shaking in a water bath WNB-22 (Memmert, Schwabach, Germany). Samples were placed in microtubes and were taken at 0.5, 1, 2, 3 and 4 h for DLS analysis without dilution. After 24 h of incubation, samples were centrifuged 5 min at 9500× *g*, and the supernatant was quantified by HPLC after dilution in methanol (1/10 or 1/2). The particle size distribution into the supernatant was also determined by DLS, using an ultra-low volume cuvette. All assays and measurements were performed in triplicate.

##### Tegaserod Content Analysis by High-Performance Liquid Chromatography (HPLC)

A C_18_/HPLC method was developed and adapted from quantitative analysis of tegaserod described by Carrier et al. and Yang et al. [45,46]. The HPLC apparatus comprised an Agilent^®^ 1290 Infinity binary pump, an Agilent 1290 Infinity autosampler and an Agilent 1260 Infinity diode-array detector (Agilent technologies, Santa Clara, CA, USA). The system was managed by ChemStation (Agilent technologies, Santa Clara, CA, USA). A reversed phase column C_18_ (5 µm, 2.1 × 50 mm, Restek^®^ Ultra, Lisses, France) was used and maintained at 40 °C during analysis. The mobile phase consisted of acetonitrile (mobile phase A) and water containing 0.1% (*v*/*v*) formic acid (mobile phase B) delivered at a flow rate of 0.4 mL/min on an elution gradient: time 0 min, A = 20%, B = 80%; time 1.5 min, A = 90%, B = 10%, maintained up to 3 min. The injection volume was set up at 1 µL, with samples diluted 1/100 in methanol and the detection wavelength was set up at 314 nm. Calibration curve was composed of seven points between 3.5 and 165 µM. The detection limit and quantification limit were 1.52 and 4.60 µM, respectively.

During stability study, several samples were diluted at 1/500 or 1/1000. Due to the lower concentrations of these samples, another calibration range was used [0.8–20 µM], and the injection volume was set up at 8 µL. For this calibration curve, the detection limit and quantification limit were 0.60 and 1.83 µM, respectively.

The detection and quantification limits were evaluated with the standard deviation of the response and the slope, according to ICH Q2(R1) standards [47]. Linearities were good within the concentration ranges studied with a correlation coefficient always higher than 0.99.

The encapsulation efficiency (EE) of tegaserod into nanoemulsions was determined after filtration through 0.2 µm syringe filters to remove unentrapped tegaserod and was calculated according to Equation (1).

(1) $$EE\left(\%\right)=100\times \frac{Quantity\;of\;tegaserod\;entrapped}{Total\;quantity\;of\;tegaserod\;added}$$

#### 2.2.3. Functionalization of Tegaserod-Loaded Nanoemulsions with Peptide-22

##### Determination of Peptide-22 Critical Aggregation Concentration

The critical aggregation concentration (CAC) of peptide-22 was analyzed, using pyrene as a fluorescent probe. Then, 6 μL of pyrene stock solution in acetone (50 μM) was added into tubes. Then, acetone was evaporated in dark conditions. Different peptide-22 suspensions ranging from 5.7 to 5741 μM were prepared in 60 mM phosphate buffer (pH 7.2). These suspensions were added into tubes and mixed overnight, at 37 °C. The pyrene final concentration was 1 μM. After 30 min of equilibration at room temperature, 200 µL of peptide-22 suspensions was transferred to 96-well plates (Greiner flat bottom black polystyrol plate, non-binding). A fluorescence spectrophotometer (Infinite M200, Tecan, Männedorf, Switzerland) was used to measure the fluorescence intensities of pyrene at an excitation wavelength of 336 nm. The emission spectra were recorded in the range 360–500 nm. The ratio of pyrene vibronic band intensities I_376_/I_382_ (I_1_/I_3_) was plotted and fitted by a Boltzmann-type sigmoid (see Supplementary Materials Figure S1). The CAC of peptide-22 was obtained as the first sharp decrease. At the same time, the hydrodynamic diameter and the particle size polydispersity index (PDI) of each suspension were determined by using DLS in triplicate, at 25 °C.

##### Peptide-22 Adsorption by Incubation with NEs Suspension

After their characterization, 500 µL of tegaserod-loaded NEs (excipients concentration of 220 µM) was incubated with the powdered peptide-22 (final concentration of 2 or 6 mg/mL), at 25 °C, in a water bath, with a stirring rate of 250 rpm. Thanks to preliminary works, the incubation time was optimized and set at 3 h. The NEs/peptide-22 mixtures were then characterized by DLS and HPLC/SEC or C18.

##### Study of Nanoemulsions/Peptide-22 Interactions through Isothermal Titration Calorimetry (ITC)

Isothermal titration calorimetry measurement was performed on a MicroCal PEAQ-ITC calorimeter (Malvern Instruments, Worcestershire, UK). A typical titration involved 40 injections of the peptide-22 (the titrant) (0.9 μL aliquot per injection, 37.3 mM) at 200 s intervals into the sample cell (volume 200 µL) containing NE suspensions (concentrations, 200 mM). The titration cell was stirred continuously at 500 rpm, and the experiment was performed at 25 °C. Blank values were determined in control titrations by injecting the peptide-22 into the buffer. To accurately quantify the thermodynamic events associated with interactions, the background signals were subtracted from raw data, and the heat generated during titration was calculated.

##### Quantification of Peptide-22 Adsorption

After identifying the optimal incubation time, a purification step was performed in order to remove the excess of free peptide in the suspension by ultrafiltration centrifugation method. Briefly, NEs/peptide-22 mixtures were centrifuged through 100 kDa Amicon^®^ Ultra filters for 15 min, at 9500× *g* and 25 °C. The filtrate volume was measured by weighing, and the retentate was resuspended in this volume of fresh phosphate buffer. The NEs/peptide-22 mixtures before ultrafiltration centrifugation, the retentate and the filtrate were analyzed by DLS. Samples were analyzed before and after purification step, using SEC/UPLC technique and C18/UPLC.

The total peptide-22 concentration represents the free and adsorbed peptide-22 in the sample. It was quantified by high-performance liquid chromatography, using the same apparatus and reversed C18 phase column that were described in Section 2.2.2. The mobile phase was composed of a mixture of 0.1% (*v*/*v*) TFA and 20% (*v*/*v*) water in acetonitrile (mobile phase A) and water containing 0.1% (*v*/*v*) TFA (mobile phase B). The gradient elution is described in Table 1. The injection volume was set to 8 µL and the detection wavelength was fixed at 210 nm. The calibration curve, ranged from 2.5 μg/mL to 100 μg/mL, was prepared by diluting the peptide-22 in methanol. The determination of the total peptide-22 concentration was achieved after dissolving 10 μL of NEs/peptide-22 mixtures into 990 µL of methanol before and after the purification step. The detection limit and quantification limit were 1.7 and 5.1 µg/mL, respectively. Linearity was good within the concentration ranges studied with a correlation coefficient always higher than 0.99.

The adsorbed peptide-22 part was obtained by measuring the free peptide-22 in the sample. The free peptide-22 concentrations were measured before and after purification step, using the high-performance liquid chromatography system/size-exclusion chromatography (UPLC/SEC) technique with a specific SEC column that allows for the separation of protein mixtures. To this end, the UPLC system aforementioned in Section 2.2.2 was used (Agilent technologies, Santa Clara, CA, USA). The column was an XBridge ProBEH SEC (125Å, 2.5 μm, 7.8 mm × 150 mm) with an XBridge ProBEH SEC Guard Column (125 Å 2.5 μm 4.6 × 30 mm, Waters). The separation between decorated NEs and free peptide-22 was carried out at 25 °C, under isocratic conditions (mobile phase: 60 mM phosphate buffer at pH 7.2), with a flow rate of 0.6 mL/min. The injection volume was set to 6 µL, and the detection wavelength was fixed at 210 nm. The NEs suspensions, the peptide-22 and the NEs/peptide-22 mixtures after incubation were diluted in phosphate buffer prior to the analysis. Free peptide concentrations were quantified by using the area under the peak in comparison with the concentration of known peptide-22 standards.

The number of peptide-22 molecules/nm² was obtained by using the volume of the particle and particle concentration (Equation (2)). These features are calculated from the data obtained by multi-angle dynamic light scattering (MADLS), assuming that homogeneous spheres were obtained. The measurements were performed with clear four-sided cuvettes, in triplicate, at 25 °C, after dilution to 1/100 in 1 mM NaCl solution with a fixed scattering mean count rate of 200 kcps.

The number of peptide-22 molecules/nm² was calculated according to Equation (2).

(2) $$P22\;(\mathrm{molecules}/\mathrm{nm}²)=\frac{\frac{{\left[P22\right]}_{\mathrm{mol}/\mathrm{mL}}\;\times \;N_{A}}{{\left[\mathrm{particules}\right]}_{\mathrm{part}/\mathrm{mL}}}}{4\pi r²}$$

##### Adsorption Stability under Dilution

The adsorption stability under dilution (1/10 and 1/100) with 60 mM phosphate buffer at pH 7.2 was assessed by measuring free peptide concentration by HPLC/SEC (method described below). Moreover, the hydrodynamic diameter and PDI were determined after each dilution, using DLS and cumulant analysis.

#### 2.2.4. Biopharmaceutical Assessment

##### In Vitro Tegaserod Release Study

The commonly used in vitro technique dialysis bag method [48] was used to study the drug-release kinetics from nanoemulsions. A total of 1 mL of tegaserod-loaded nanoemulsions was instilled into a cellulose ester dialysis bag (Spectra/Por^®^ Biotech membranes, molecular weight cutoff 100 kDa, Spectrum Laboratories, Rancho Dominguez, California, USA) and incubated in PBS (European pharmacopeia, 10th ed.), pH 7.4, in a water bath WNB-22 (Memmert, Schwabach, Germany) at 37 °C, under gentle horizontal shaking. Polysorbate 80 (1%, *v*/*v*) was added to the acceptor compartment to satisfy sink conditions. Drug release was analyzed by removing of 1mL aliquots at appropriate intervals, and replaced with fresh PBS. The amount of tegaserod released at each time was measured by HPLC, considering the cumulative quantity removed. All measurements were performed in triplicate.

##### In Vitro Hemolysis Assay

Hemolysis tests were realized according to the protocol previously described by our team [38]. Briefly, whole human blood samples from three healthy compatible volunteers were collected in Li-heparin tubes (Etablissement Français du Sang, EFS Hauts-de-France-Normandie, France), pooled and diluted in DPBS to adjust the total hemoglobin concentration at 10 mg/mL. For each test, 100 µL of sample was introduced into microtubes with 700 µL of DPBS and 100 µL of diluted blood. Tegaserod suspension or tegaserod-loaded nanoemulsions, with and without adsorbed peptide-22, were incubated with blood at the final concentration range of tegaserod 0.5–23.0 µg/mL, during three hours, at 37 °C, with constant horizontal shaking (water bath WNB-22). After incubation, the samples were centrifuged (15 min at 850× *g*, 25 °C, using a Universal 320R apparatus, Hettich, Bäch, Switzerland), and the hemoglobin released during the hemolysis test was quantified by spectrophotometry (Infinite M200, Tecan, Männedorf, Switzerland) by determining the absorbance of red cyanmethemoglobin (CMH), at 540 nm, in a 96-well plate.

These measured absorbances were compared to a standard curve of human hemoglobin with satisfactory linearity (R^2^ > 0.99) in the concentration range studied (0.0625–1 mg/mL). The results shown for the nanoemulsions were adjusted by taking account of the eventual interferences of nanoemulsions at 540 nm.

In this assay, a hemolysis threshold of 5% was defined. When it was crossed, the compound was considered hemolytic. All assays were performed in triplicate.

#### 2.2.5. Statistical Analysis

Statistical analyses were performed by using the Prism 6.05 software (Prism Software, Irvine, CA, USA). The non-parametric Mann–Whitney test was used, and a *p* < 0.05, at least, was considered statistically significant.

## 3. Results

### 3.1. Tegaserod Characterization

#### 3.1.1. Tegaserod Pharmacological Characterization

The cholinergic hypothesis is the oldest theory in the AD pathogenesis explanation based on cholinergic neurons loss and acetylcholine (ACh) deficit, directly linked to cognitive decline. Considering that ACh is hydrolyzed by both AChE and BuChE, tegaserod was evaluated as a potential inhibitor of human AChE and equine BuChE, using Ellman’s assay [42]. It was compared to donepezil and tacrine, and used as reference AChE and BuChE inhibitors, respectively (Table 2). Tegaserod was shown to be able to inhibit BuChE with IC_50_ value in micromolar range, whereas it displayed a negligible AChE inhibitor activity.

Of course, tegaserod is known as 5-HT_4_R agonist. Its strong binding affinity toward 5-HT_4_ receptors was estimated with a p*K*i around 8.0 [49,50]. Therefore, from these pharmacological data, tegaserod should present a dual pharmacological activity as 5-HT_4_R agonist and, to a lesser extent, as BuChE inhibitor. In healthy brains, AChE activity predominates and BuChE plays a minor role in the brain metabolism of ACh [51]. On the contrary, in patients with progressed AD, a decrease level of AChE activity was demonstrated with the progressive increase of BuChE activity, which mostly hydrolyzed the ACh [52]. In consequence, the BuChE inhibition activity of tegaserod should be considered as a promising therapeutic effect in AD. Indeed, considering the multifactorial circumstances of the disease, 5-HT_4_Rs activation alone might not be sufficient to reverse the progression of AD, and such a combination of effects is promising [53].

**Figure 2:**
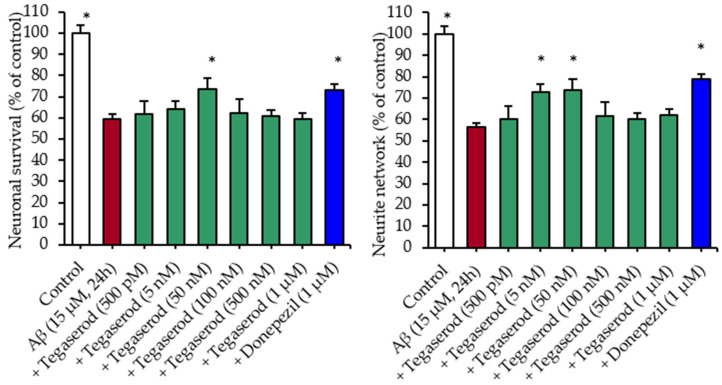
Neuronal survival and neurite network in a primary culture of cortical neurons injured with Aβ. * *p* < 0.05 versus Aβ_1–42_.

Several 5-HT_4_R agonists (RS67,333, donecopride) have been proven to show beneficial effects on learning and memory loss in 5XFAD mouse model, with a reduction of the number of observed amyloid plaques [5,54]. In regard to these results, the effects of tegaserod on neuronal survival and on the neurite network were investigated in rat primary hippocampal neurons exposed for 24 h to soluble Aβ_1–42_ peptides. As previously reported [54], the application of the soluble Aβ peptides led to a significant loss of neurons and to an important neurite network reduction (Figure 2). Donepezil, used here as a positive control, displayed a neuroprotective effect and was able to preserve the neurite network. As with donepezil, but at a lower required concentration (50 nM vs. 1 µM), tegaserod appeared to be able to protect neurons from death, and exerted also protective effects on the neurite network at the low tested concentrations of 5 and 50 nM. Last, as already observed for another promising drug for AD, donecopride, acting not only as an AChE inhibitor and a 5-HT_4_R agonist [55], high doses of agonists could induce the downregulation of 5-HT_4_ receptors.

#### 3.1.2. Tegaserod Physicochemical Characterization

Some physicochemical properties of tegaserod, correlated to its drugability profile, were defined (Table 3). First, tegaserod can be considered as a lipophilic molecule, regarding its log*P* value and its thermodynamic solubility at pH 7.4. Due to its ionizable groups, tegaserod exhibited three p*Ka* between 1.94 and 15.24 and a pH-dependent solubility. At physiological pH, tegaserod behaves as a weakly alkaline drug with greater solubility at pH < p*Ka* [56]. Thus, this alkaline lipophilic drug is highly soluble at acidic pH, and its solubility decreases gradually between pH range 2 and 8. A consequence of such a profile may be, for example, hampering of its solubility in blood compartment. It is also in good agreement with previous data of clinical development, where tegaserod was found to be 98% bound to plasma proteins in human plasma, especially to α_1_-acid glycoproteins, which carry a basic charged lipophilic compound [57].

Being largely proposed in HTS approaches, a PAMPA model was used to predict the passive gastrointestinal absorption of tegaserod (Table 3). Tegaserod was characterized as not able to passively diffuse through the GIT biomimetic membrane, regardless of the pH in the donor compartment. Hasler and Schoenfeld noted that tegaserod has shown a rapid absorption from the small intestine [58]. Regulatory agencies affirmed that tegaserod exhibited an oral absorption under 35% and an oral bioavailability about 10% [59,60]. In consequence, tegaserod would be actively transported, and our PAMPA-GIT results would be misinterpreted.

The ability to cross the BBB by using a PAMPA experiment was also assessed. Noticeably, the nature of the lipid mixtures used in both PAMPA models is different to mimic the BBB and GIT membranes. Through BBB, tegaserod would have a moderate permeation potential with Pe = 81.9 ± 13.2 nm s^−1^. Due to its peripheral indication and its innovative repurposing in AD, no BBB permeability value of tegaserod was given in the literature. However, the FDA reported six non-clinical studies in rodents, realized for tegaserod approval after oral, intravenous and intracarotid administration. In these reports, tegaserod was not detected or detected at very low appreciable concentration into the brain [14]. Its negligible penetration is due in part to its high plasma-protein binding and its positive charge at physiological pH [57,61], as outlined by Appel-Dingemanse. These results highlighted that tegaserod would not cross the BBB to any appreciable extent at therapeutically relevant doses. This is all the more exact that tegaserod would be a p-glycoprotein (Pgp) substrate [59,62]. In this case, PAMPA–BBB tends to overestimate the permeability of the drug, due to active processes [63].

#### 3.1.3. Tegaserod Profile

From the neuroprotective cellular effects of tegaserod, highlighted in the present study, this drug should be repurposed in order to provide some disease-modifying effects of great interest in AD treatment. Nevertheless, to succeed in such a drug repurposing, it is crucial to consider, as early as possible, the best formulation to further preclinical and clinical studies.

The development of an adapted formulation for intravenous route seems particularly appealing to the establishment of the proof-of-concept regarding the pharmacological interest of tegaserod in AD treatment. In regard to its low solubility, its moderate permeability and also its potential side effects, the encapsulation of tegaserod into safe lipid-based nanocarriers could be a valuable option to improve its biodistribution and to ensure its safety profile.

Indeed, the human ether-à-go-go-related gene (hERG) potassium channel is known to play a pivotal role in regulating cardiac excitability and maintaining normal cardiac rhythm. It is, moreover, a target of many drugs that cause the acquired form of prolonged QT syndrome [64]. According to the bibliographic data, the mechanism basis of the described cardiovascular side effects of tegaserod remains unknown, and interactions with hERG potassium channel or 5-HT_1_ receptor subtypes have been suspected to cause myocardial infarction. From our in vitro results, we noted that tegaserod exhibited weak hERG potassium channel affinity associated with an IC_50_ of 3020 nM (see Supplementary Materials Figure S2). Moreover, FDA reports mentioned a negligible binding affinity with an IC_50_ value for tegaserod of 13,000 nM on cloned hERG channels expressed in HEK 293 cells. This value is very high compared to the IC_50_ of cisapride around 44 nM obtained in same conditions [60]. This is coherent with the fact that tegaserod was not associated with a QT interval prolongation [65,66]. However, tegaserod is a 5-HT_1B/D_ subtypes receptors agonist. These receptor agonists are known as “triptans” used for migraine headache treatment associated with rare cardiovascular adverse events. Chan et al. have studied tegaserod induced contractions on isolated coronary arteries and they concluded that tegaserod did not show vasoconstrictor potential at clinical relevant concentrations [67]. To avoid side effects, encapsulation of tegaserod into nanocarriers appears very appealing, all the more with the final aim to carry tegaserod to the CNS after intravenous administration.

### 3.2. Encapsulation of Tegaserod into Nanoemulsions

#### 3.2.1. Characterization of Nanoemulsions

Among lipid-based nanocarriers, nanoemulsions present many advantages. They consist of oil nanodroplets dispersed in an aqueous phase. Nanoemulsions are particularly suitable for encapsulation of low soluble molecules into their oily core. They permit to obtain high loading efficiency. Thanks to well-adapted excipients, they may be also non-toxic, highly stable and easily produced, particularly by low energy processes [38,68].

**Figure 3:**
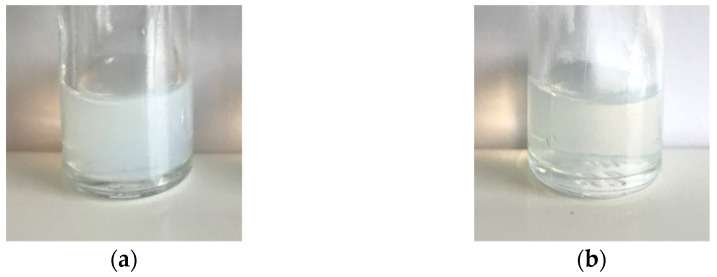
Macroscopic observation of Blank-NEs (**a**) and Tg-NEs (**b**).

The nanoemulsion formulation, and the same spontaneous emulsification process, previously described by our team [38], was transposed to tegaserod. After formulation, the obtained tegaserod-loaded nanoemulsions (Tg-NEs) had a clear appearance with a slight greenish coloration attributable to tegaserod (Figure 3). The Tg-NEs were characterized in terms of granulometric properties, physicochemical properties and efficacy of encapsulation (Table 4). The encapsulation of tegaserod succeeded with an encapsulation efficacy higher than 90% at a drug payload of 1 mg/mL. The hydrodynamic diameter slightly decreased. PDI was not changed relative to the blank-NEs, since remaining lower than 0.2, indicating the presence of a monodisperse population. However, the incorporation of the tegaserod into the NEs was characterized by a slight but significant increase of ζ-potential (*p* < 0.0001). This observation was consistent with the p*Ka* values of tegaserod and the fact that its mono protonated form was predominant at pH 7.4. In this way, these results indicated that a part of tegaserod could be located at the oil/water interface of nanoemulsions.

Moreover, thanks to an original and suitable phosphate buffer as the aqueous phase, the pH and osmolarity of Tg-NEs were well suited for intravenous administration (Table 4).

#### 3.2.2. Stability Study of Tg-NEs over Time

**Figure 4:**
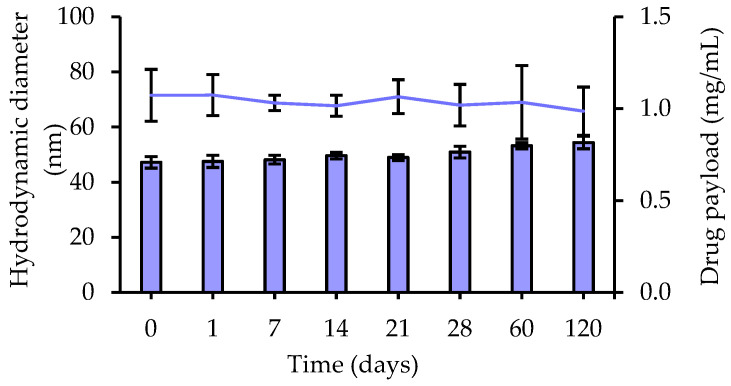
Stability of Tg-NEs estimated by the hydrodynamic diameter (bars) and the drug payload (line) during 120-day storage at 4 °C.

The storage stability of undiluted Tg-NEs was assessed at 4 °C for 4 months, and the macroscopic aspect, hydrodynamic diameter, PDI, ζ-potential and drug payload were regularly monitored. No significant change of these physical-stability indicators was observed up to 21 days, revealing a good stability of Tg-NEs under storage conditions at 4 °C (Figure 4). A slight and gradual increase of the hydrodynamic diameter occurred between the 21st and the 120th days (Δ = +7 nm) without any change of the PDI, nor of the drug payload. Although nanoemulsions are thermodynamically unstable systems that are frequently the subject of destabilization over time by creaming, sedimentation, flocculation, coalescence or Ostwald ripening [69], the present nanoemulsions were designed to have a kinetic stability. The encapsulation of tegaserod and the slight ζ-potential increase, remaining, however, close to neutral values, did not disturb the nanoemulsions stability previously observed for blank-NEs, probably thanks to steric stabilization [38].

**Figure 5:**
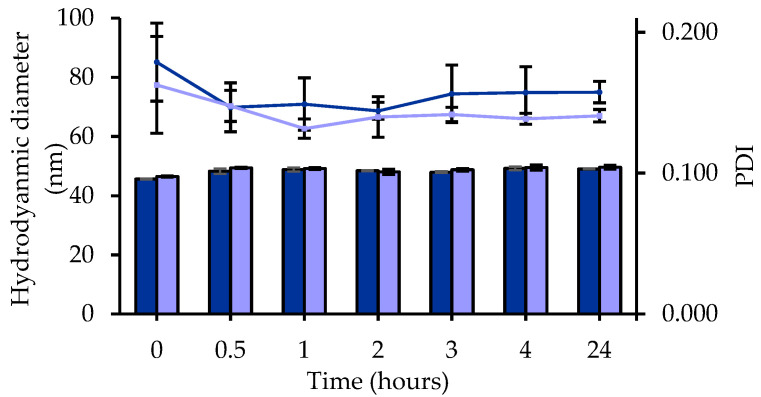
Stability profile of Tg-NEs estimated by the hydrodynamic diameter (bars) and the polydispersity index (line) in PBS pH 7.4 after 1/100 (dark blue) and 1/500 (light blue) dilutions (*n* = 3, data are shown as the mean ± SD).

Nanoemulsions’ stability in biomimetic media is another important factor to consider before preclinical studies, particularly if the formulation is intended for intravenous route. Indeed, nanoemulsions may present some instability upon dilution by injection in the bloodstream. Therefore, a stability study at 37 °C was performed in PBS as plasma biomimetic media after 1/100 and 1/500 dilutions. Tg-NEs were found to be stable and homogenous under dilution and body temperature, with no appreciable changes over the time in terms of hydrodynamic diameter, PDI and drug payload for 24 h (Figure 5). These results predicted a good stability of the nanoemulsions in the bloodstream, especially since tegaserod has been shown to remain stable in these conditions [70]. The nanoemulsions integrity could be preserved to reach the BBB.

As described in the literature [71,72], Kolliphor^®^ HS 15 is a known inhibitor of P-gp activity. In light of these data, we could hope that tegaserod encapsulation into our nanoemulsions containing Kolliphor^®^ HS 15 would improve its BBB permeation. In order to further increase the BBB crossing of tegaserod-loaded NEs, we aimed at decorating nanoemulsions with peptide-22, a peptide with special affinity for low-density lipoprotein receptors.

### 3.3. Functionalization of Tegaserod-Loaded Nanoemulsions with Peptide-22

Using phage display biopanning directed toward the human LDL-Rs, Malcor et al. isolated and then optimized one new ligand with high LDL-Rs-binding affinity: peptide-22 [39]. Peptide-22 is a cyclic peptide with reduced peptide bonds and one non-natural amino acid. With a half-life of about 3 h, this peptide exhibits no competition with endogenous LDL [39]. This short half-life is attributed to protease degradation. The adsorption method may confer a protection against proteolytic degradation, due to PEG on the surface of NEs, which provided steric hindrance and thus blocked interactions between peptide and enzyme [73]. Several studies have been carried out on nanocarriers, i.e., polymeric nanoparticles [40], liposomes [41], functionalized by peptide-22 for glioma treatment. Reported in vitro and in vivo studies confirmed the beneficial effect of such functionalization with significant enhanced brain permeation, stronger chemotherapeutic effect and enhanced mice survival rate in glioma mouse models [40]. Therefore, we proposed to perform peptide-22 adsorption on Tg-NEs in order to target the BBB and enhance the delivery of tegaserod to the CNS.

#### 3.3.1. Peptide-22 Adsorption on Tg-NEs

In their review of relevant strategies to functionalize lipid-based nanocarriers, Guyon et al. mentioned four methods: (i) the direct ligand conjugation on preformed nanocarriers, (ii) the post-insertion of PEG-ligand on preformed nanocarriers, (iii) the incorporation of PEG-ligand during the formulation process and (iv) the noncovalent adsorption of the ligand at the surface of nanocarriers [23]. This last method has already demonstrated its advantages with good adsorption efficiency and preservation of peptide activity [73]. To avoid any possible loss of peptide activity after its covalent link to any PEG moiety, and because of its ease of use and being very fast, we have chosen the peptide adsorption method as valuable option in our formulation assays.

The adsorption experiments were realized with two peptide initial concentrations, i.e., 2 mg/mL (Tg-NEs-P22_2_) and 6 mg/mL (Tg-NEs-P22_6_) equal to a peptide/excipients molar ratio of 1:114 and 1:38, respectively. In case of Tg-NEs-P22_6_, the molar ratio is similar to the one used by Chen et al. of 1:31 [41]. At both concentrations, peptide-22 in 60 mM phosphate buffer self-organizes in relatively monodisperse nanostructures (PDI < 0.250) with an average hydrodynamic diameter of ∼2 nm (see Supplementary Materials Figure S1).

As shown in Table 5, adsorption process did not disturb the stability of the Tg-NEs whatever the concentration of the peptide. Indeed, the conjugation of Tg-NEs with the peptide-22 did not alter their hydrodynamic diameter and monodispersity. However, as already observed by Chen et al., the presence of significant amounts of peptide-22 on the surface of the nanodroplets, in case of Tg-NEs-P22_6_, increased slightly but significantly the zeta potential (*p* < 0.05) [41].

Isothermal titration calorimetry (ITC) assays have been carried out to objectify and further characterize the peptide-22/Tg-NEs interactions. Indeed, this label-free and sensitive technique, which is based on heat measurement absorbed or generated during binding event in a titrating manner, appears as a method of choice to provide a complete thermodynamic profile of such biomolecule–nanocarrier interactions [23,74,75]. To ensure small background heats of dilution that would affect binding measurements, the native Tg-NEs formulation was placed into the sample cell, and titration by peptide-22 up to a final concentration of 6 mg/mL was performed. From the curve for NEs titration with peptide-22, it can be seen that the reaction is exothermic (see Supplementary Materials Figure S3), indicating biomolecular interactions involving non-covalent bond formation processes (van der Waals forces, electrostatic interactions or hydrogen bonds) [74]. Nevertheless, in the used molecular concentration range, that was similar to that implemented during the adsorption experiments, it was not possible to achieve binding saturation, making it impossible to extract any quantitative thermodynamic information such as binding stoichiometry, binding constant, enthalpy and entropy change from the non-optimal titration curve. Obviously, other concentration ranges should have been assayed by keeping unchanged the peptide-22/Tg-NEs ratio. Nevertheless, the lower concentration range did not generate measurable heat signals (results not shown), or higher ones could not be performed, as they required material quantities that were too high.

Besides, to characterize adsorption of peptide-22 on Tg-NEs, the peptide-22 concentration was determined by C_18_/HPLC after the separation of free and adsorbed peptide-22 by the commonly used ultrafiltration centrifugation method through Amicon^®^ Ultra filters devices. Prior to assays, it was checked on peptide-22 alone at 2 or 6 mg/mL in phosphate buffer, that all the peptide was recovered in the filtrate after a 9500× *g* centrifugation for 15 min. The peptide-22 adsorption on Tg-NEs was measured by retentate dosing after resuspension in phosphate buffer and the total recovery of peptide-22 was guaranteed by the filtrate dosing. With this method, the peptide-22 adsorption efficiency was 23% and 43% for Tg-NEs-P22_2_ and Tg-NEs-P22_6_, respectively.

To bypass some possible limitations of ultrafiltration centrifugation devices, we proposed to check this result by additional techniques. Indeed, from recent studies reported in the literature, lipid nanocapsules of 50 nm diameter could pass through the Amicon^®^ filter, clog it and thus skewing the method [76]. Moreover, the centrifugation step itself could be responsible for peptide desorption. In consequence, an innovative chromatography exclusion analysis (SEC/HPLC) was used to separate, in the course of the analysis and without prior purification step, the decorated nanodroplets from smaller molecules, i.e., the free peptide-22 molecules (see Supplementary Materials Figure S4). The free fraction of peptide-22 was quantified to allow the indirect determination of the adsorbed fraction on the surface of the Tg-NEs. It was determined by SEC/HPLC on native Tg-NEs-P22 and on the retentate, after the ultrafiltration centrifugation process. In both cases, results obtained by the SEC/HPLC dosing method were similar to those obtained by C_18_/HPLC (Table 5). Thus, the purification of samples by ultrafiltration centrifugation seemed suitable to these developed nanoemulsions. From both dosing methods, the higher concentration of peptide-22 used allowed a better success rate of 40 ± 8% and thus, the adsorption of a greater amount of peptide-22 molecules per nanodroplet of about 680 ± 140 after purification.

#### 3.3.2. Adsorption Stability of the Peptide-22 under Dilution

The adsorption stability of peptide-22 under dilution in PBS was assessed since these nanoemulsions are intended to be administered intravenously, and could present some instability during injection in the bloodstream (Table 6). The amount of adsorbed peptide-22 was found to be stable after dilution in case of Tg-NEs-P226, in contrast to Tg-NEs-P222 for which the amount of adsorbed peptide decreased from 0.015 to 0.006 molecules per nm². Even in these conditions, such a density of peptide-22 at the NEs surface of 0.006 molecules per nm² matches with the density of peptide-22 obtained in other studies. Indeed, it was reported that a density of 0.005 molecules of peptide-22/nm² of nanoparticles significantly enhanced brain permeation of the nanoparticles compared to undecorated nanoparticles [40]. Consequently, such Tg-NEs appear a valuable option for future preclinical studies.

To optimize targeting to the LDL-Rs, and hence to carry tegaserod to the CNS after intravenous administration, Tg-NEs should be functionalized with the greatest number of peptide molecules. Moreover, to discard any saturation of the receptors by free peptide molecules and favor transcytosis of the Tg-NEs, it is essential to obtain a purified and stable formulation. In consequence, we decided to use an initial concentration of peptide of 6 mg/mL for further studies of the Tg-NEs-P22. Such Tg-NEs-P22 appear suitable for intravenous administration since the formulation was shown to present a pH of 7.3 ± 0.1 and an osmolarity of 284 ± 20 mOsm. Moreover, the encapsulation efficiency of tegaserod stayed stable after adsorption and purification steps (88 ± 13%).

### 3.4. Biopharmaceutical Assessment of Tg-NEs and Tg-NEs-P22

#### 3.4.1. In Vitro Release of Tegaserod

**Figure 6:**
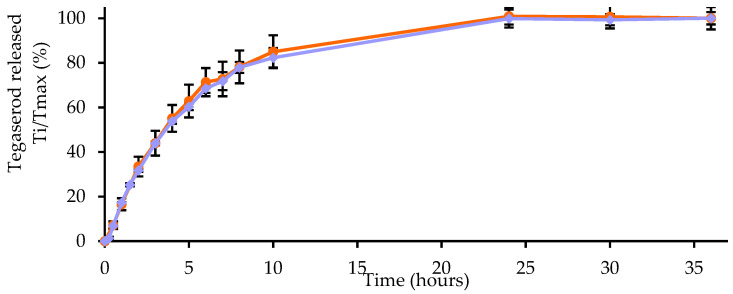
Tegaserod release profile from Tg-NEs (in blue) and Tg-NEs-P22 (in orange) in PBS pH 7.4 (*n* = 3, data are shown as the mean ± SD).

Tg-NEs achieved approximately 80% of drug release within 10 h, followed by a slower release within 24 h (Figure 6). Regarding the Tg-NEs-P22, the curves were similar suggesting that the adsorption of peptide-22 did not evidently affect the in vitro release behavior of tegaserod from nanoemulsions. At the end of the assay, each compartment was analyzed by DLS. Nanoemulsions were found unchanged in terms of hydrodynamic diameter and PDI into the dialysis bag. However, the ζ-potential decreased to reach the value of blank-NEs. The nanoemulsions droplets were not found in the acceptor medium where only micelles of polysorbate 80, used to maintain sink conditions, were present.

Interestingly, no burst release was observed for both formulations predicting very few, if any side effects after administration. Recently, Fan et al. have shown that nanoemulsions formulated with Labrafac^®^ WL 1349 and Solutol^®^ HS 15, measuring 70 nm in diameter, rapidly reached the brain within a few hours after intravenous administration (from 1 h) [77]. Moreover, Zhang et al. have demonstrated that the functionalization of nanoparticles allowed a higher brain content up to 10 h compared to undecorated nanoparticles [40]. Thus, encapsulation of tegaserod in nanoemulsions appears to be a promising strategy to reach the brain, particularly after functionalization by peptide-22.

#### 3.4.2. In Vitro Hemolysis Assay

Last, before undertaking any in vivo pharmacokinetics and pharmacodynamics studies, which will be the object of a further work, it appeared essential to evaluate the compatibility of the nanocarriers with red blood cells in regard to the intravenous administration route, that will be intended to use. As shown in a previous work of our lab [38], blank-nanoemulsions are considered non-hemolytic, but tegaserod encapsulation could change their safety profile. In this study, the hemolysis assay on whole human blood was applied to tegaserod suspension compared to Tg-NEs, themselves compared to the results of blank-NEs. Different concentrations of tegaserod were tested, free or encapsulated, as well as the respective blank-NEs.

**Figure 7:**
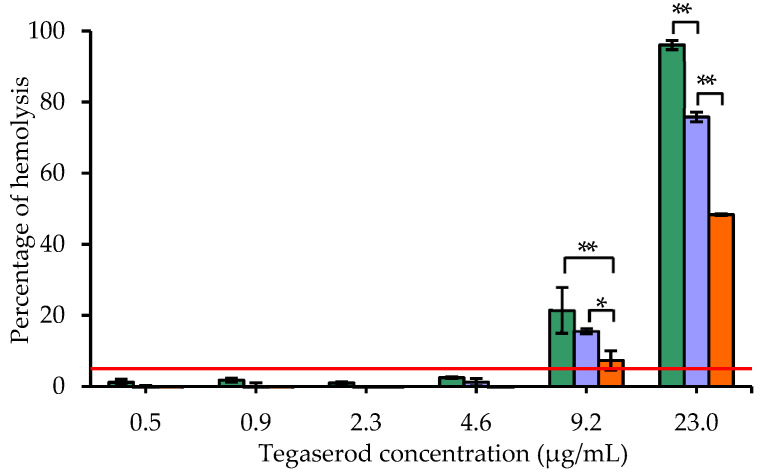
Results of the hemolysis test for the suspension of tegaserod (in green), Tg-NEs (in blue) and P22-Tg-NEs (in orange). The red line represents the threshold value of 5%. Note: Data are shown as mean ± standard deviation (*n* = 3). * *p* < 0.05; ** *p* < 0.0001.

A significant correlation between the tegaserod content and the hemolytic activity was objectified (Figure 7). At the highest concentration tested of 23.0 µg/mL, all samples with tegaserod were significantly hemolytic. Interestingly, tegaserod encapsulation into the nanoemulsions reduced significantly its hemolytic properties, itself reduced significantly by the peptide-22 adsorption on Tg-NEs (*p* < 0.0001). The same trend was observed for the concentration of 9.2 µg/mL with a significant decrease of the hemolytic activity of P22-Tg-NEs (*p* < 0.0001). It is important to note that the hemolytic activity of the nanoemulsions was measured after 3 hours’ incubation and at this time, the released tegaserod quantity from both Tg-NEs and P22-Tg-NEs was not significantly different. In consequence, the decrease in hemolytic activity observed for P22-Tg-NEs would be objectively due to the surface protection awarded by the adsorption of peptide-22.

At the concentration of 9.2 µg/mL of tegaserod, equivalent blank-NEs calculated according to the amount of excipients, were not hemolytic [38]. At the concentration of 23.0 µg/mL of tegaserod, equal blank-NEs shown only 9.9 ± 1.6% of hemolysis. This test revealed that at high concentrations Tg-NEs and Tg-NEs-P22 were significantly more hemolytic than blank-NEs (*p* < 0.0001). These data lend support to the supposed position of tegaserod at the surface of the nanodroplets. Indeed, this hemolytic activity could be explained by their cationic charge attributable to the presence of tegaserod. These cationic charges could interact with the negatively charged surface of the red blood cells, conferred by sialylated glycoproteins [78], and disturb the membrane structure by electrostatic interactions [79,80,81]. However, from 0.5 to 4.6 µg/mL the hemolysis was less than 5% for both nanoemulsions. To avoid any hemolysis after intravenous administration the concentration of P22-Tg-NEs would be locally decreased by a slow injection.

Considering that the tegaserod appeared to be ten-times more effective than donecopride on neurite networks in vitro, the dose administered in vivo could be ten times lower. In vivo, donecopride displayed anti-amnesic properties at a dose of 1 mg/kg [54]. In a mouse model, the administration of 0.1 mg/kg of tegaserod was equal to the concentration of tegaserod of 1.4 µg/mL, which is a non-hemolytic concentration [82]. In these conditions, the developed Tg-NEs-P22 formulation appears well adapted for further pharmacokinetic and efficacy studies.

## 4. Conclusions

In view of the lack of therapeutic treatment for Alzheimer’s disease, new therapeutic strategies are primordial. Considering both butyrylcholinesterase inhibition activity and neuroprotective properties of tegaserod that have been shown in the present work, the repurposing of this 5-HT4R agonist should be a valuable option for AD treatment. To circumvent its poor drugability profile, biocompatible tegaserod-loaded nanoemulsions were successfully formulated and functionalized by the brain-targeting peptide-22 in order to optimize its biodistribution towards CNS, while limiting any adverse cardiac effect. Such active targeting should increase the chance of success moving the repurposed drug through the different preclinical and clinical phases.

## Figures and Tables

**Table 1:** Gradient elution for total peptide-22 concentration analysis.

Time (min)	Flow (mL/min)	Phase A (%)	Phase B (%)
0	0.6	10	90
0.2	0.6	10	90
1.0	0.6	50	50
1.1	0.6	50	50
1.2	0.6	10	90

**Table 2:** Inhibitory activity of eqBuChE and hAChE (% inhibition or IC_50_).

Conpound	eqBuChE		hAChE
	% inhibition at 1.10^−5^ M	IC_50_	% inhibition at 1.10^−5^ M
Tacrine	98%	3.2 ± 0.6 nM	-
Donepezil	-	-	97%
Tegaserod	78%	4.2 ± 0.9 µM	2%

**Table 3:** Physicochemical and permeability data concerning tegaserod.

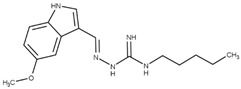	Values
Molecular weight (g/mol)	301.39
p*Ka*_1_, p*Ka*_2_ and p*Ka*_3_	1.94, 8.50 and 15.24
Log*P*	2.95
Thermodynamic solubility at pH 7.4 (µM)	151.5 ± 8.6
PAMPA gastro intestinal tract permeability assay at pH 5: Pe (nm/s)	0.0 ± 0.0
PAMPA gastro intestinal tract permeability assay at pH 6: Pe (nm/s)	0.0 ± 0.0
PAMPA gastro intestinal tract permeability assay at pH 7.4: Pe (nm/s)	0.0 ± 0.0
PAMPA blood–brain barrier permeability assay: Pe (nm/s)	81.9 ± 13.2

pKa and Log*P* (Consensus method) were calculated by using ChemAxon software Marvin 19.1.0, 2019, ChemAxon (http://www.chemaxon.com/)).

**Table 4:** Physicochemical characteristics of nanoemulsions (*n* ≥ 18).

Formulation	Hydrodynamic Diameter (nm)	PDI	Ζ Potential (mV)	pH	Osmolarity (mOsm)	EE (%)
Blank-NEs	51.6 ± 2.0	0.170 ± 0.014	−6.9 ± 2.4	7.2 ± 0.1	283 ± 10	-
Tg-NEs	47.2 ± 2.1	0.167 ± 0.015	1.8 ± 4.0	7.4 ± 0.1	312 ± 10	91 ± 10

**Table 5:** Physicochemical characteristics of nanoemulsions (*n* ≥ 3).

Physicochemical Characteristics	Tg-NEs	Tg-NEs-P22_2_	Tg-NEs-P22_6_
Hydrodynamic diameter (nm)	47.2 ± 2.1	47.8 ± 1.1	50.2 ± 3.5
PDI	0.167 ± 0.015	0.152 ± 0.018	0.147 ± 0.016
ζ potential (mV)	1.8 ± 4.0	4.8 ± 11.0	5.6 ± 1.7
Adsorption efficiency by SEC/HPLC (%)	-	20 ± 3	45 ± 4
Peptide molecules/nanodroplet	-	115 ± 15	740 ± 80

**Table 6:** Adsorption stability of Tg-NEs/P22 mixture determined with SEC/HPLC before separation under dilution in phosphate buffer (pH 7.2, 60 mM) as a function of the initial peptide 22 concentration.

Dilution Factor	Peptide Molecules/Nanodroplet			
	1:1	1:10	1:100	1:200
[P22] = 2 mg/mL	115	115	45	50
[P22] = 6 mg/mL	510	645	550	540

## Data Availability

The data presented in this study are available in [insert article or Supplementary Material here].

## Supplementary Materials

The following are available online at www.mdpi.com/1999-4923/13/10/0/s1. Figure S1: Self-assembling properties of P22 suspension in 60 mM phosphate buffer, Figure S2: hERG channel inhibition profile of tegaserod and E-4031 as reference. Figure S3: Isothermal titration calorimetry data obtained for the binding interaction in 60 mM phosphate buffer at 25 °C of P22 (37.3 mM) to Tg-NEs (200 mM). Figure S4: Characterization of P22 adsorption on Tg-NEs, using SEC coupled with UPLC.
