# Supplementary material for: Active Targeted Nanoemulsions for Repurposing of Tegaserod in Alzheimer’s Disease Treatment
Source: Pharmaceutics. 2021 Oct 6;13(10):1626. doi: 10.3390/pharmaceutics13101626 (PMC8540544; doi:10.3390/pharmaceutics13101626)
Supplement: Supplementary file 1 [file pharmaceutics-13-01626-s001.zip › pharmaceutics-1396225-supplementary.pdf]

pharmaceutics

MDPI

Supplementary Materials: Active Targeted Nanoemulsions for Repurposing of Tegaserod in Alzheimer's Disease Treatment

Line Séguy, Léna Guyon, Manon Maurel, Pascal Verdié, Audrey Davis, Sophie Corvaisier, Vincent Lisowski, Patrick Dallemagne, Anne-Claire Groo and Aurélie Malzert-Fréon

(**a**)

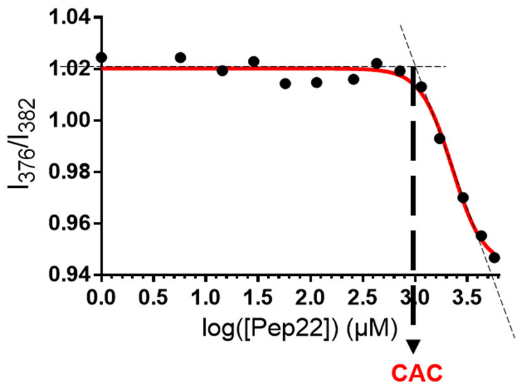

(**b**)

[P22] µM	[P22] µg/mL	*Z*-average (nm)	PDI	
14.4	15.0	701.0 ± 1206.0	0.533 ± 0.399	
258.4	270.0	632.4 ± 1079.0	0.563 ± 0.255	
				CAC
1722.5	1800.0	2.6 ± 0.8	0.176 ± 0.034	
5741.6	6000.0	2.2 ± 0.0	0.206 ± 0.007	

Figure S1. Self-assembling properties of P22 suspension in 60 mM phosphate buffer. **(a)** Boltzmann-type sigmoid obtained by the pyrene fluorescence 1:3 ratio method showing the CAC value, corresponding to the first sharp decrease. **(b)** Features of P22 suspensions at different concentrations obtained by DLS. PDI is the particle size polydispersity index. Measurements are expressed as the average ± the standard deviation.

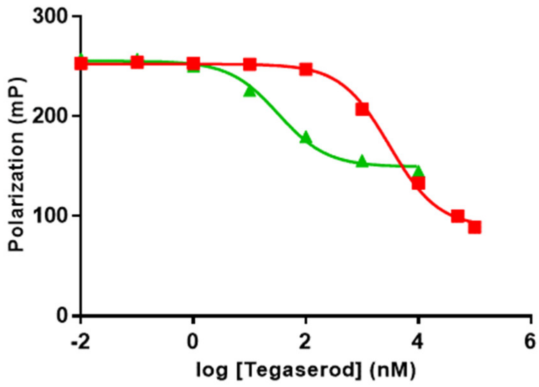

Figure S2. hERG channel inhibition profile of tegaserod (in red) and E-4031 as reference (in green).

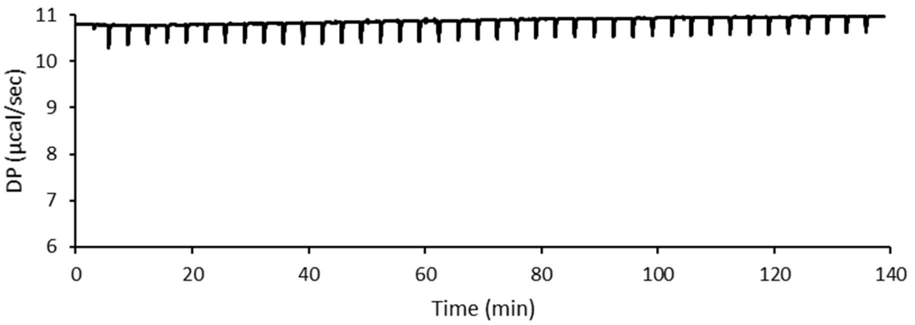

(**a**)

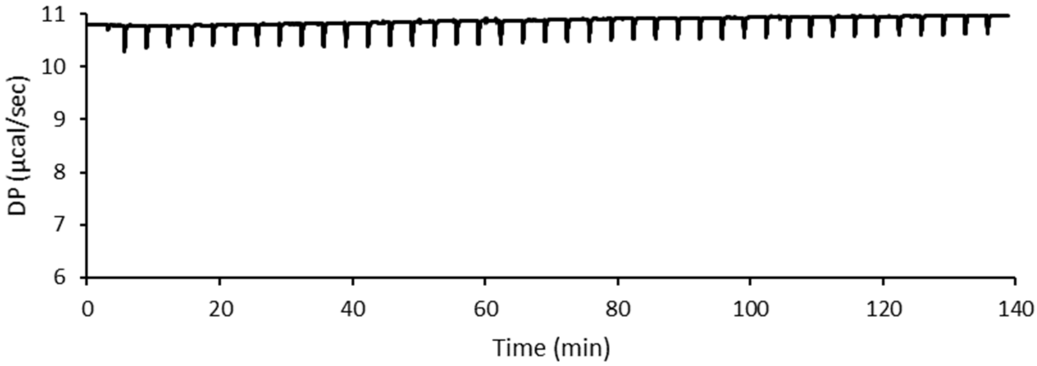

(**b**)

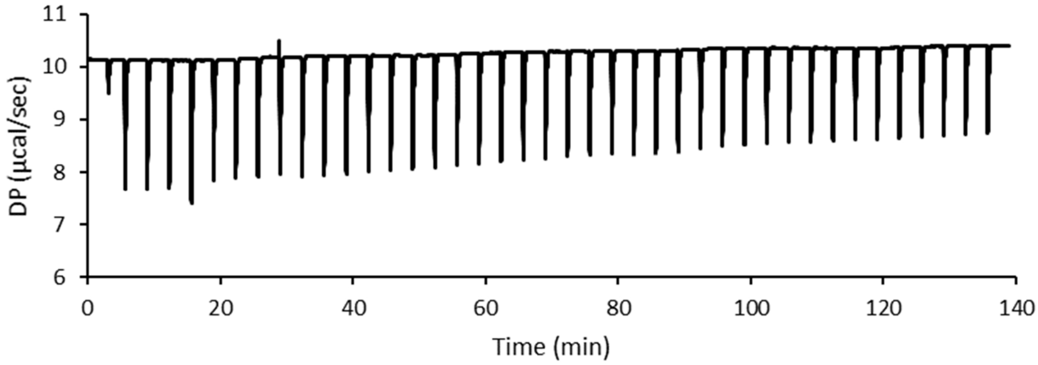

(**c**)

Figure S3. Isothermal titration calorimetry data obtained for the binding interaction in 60 mM phosphate buffer at 25 °C of P22 (37.3 mM) to Tg-NES (200 mM). (**a**) Shows the heat peptide dilution: injection of 0.9 µL aliquots of P22 solution in 60 mM phosphate buffer. **(b)** Shows exothermic heat releases upon injection of 0.9 µL aliquots of P22 solution in Tg-NEs. **(c)** Shows integrated heat data, giving a differential binding curve.

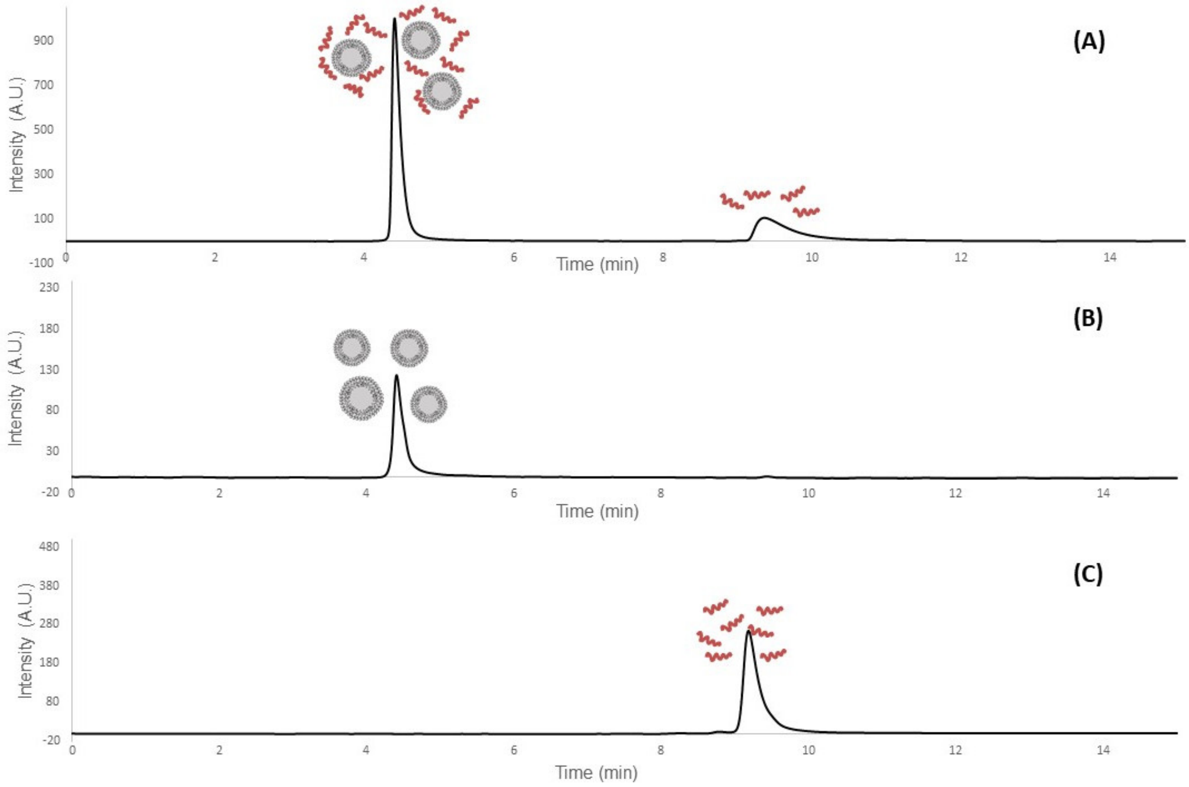

Figure S4. Characterization of P22 adsorption on Tg-NEs using SEC coupled with UPLC. Chromatograms of **(A)** Tg-NEs/P22 mixture ([P22] = 600 μg/mL, [lipid] = 20 mM) after 3 hours of incubation, **(B)** Tg-NEs ([lipid] = 2 mM) and **(C)** P22 ([P22] = 200 μg/mL).
